# X-ray ptychographic and fluorescence microscopy of frozen-hydrated cells using continuous scanning

**DOI:** 10.1038/s41598-017-00569-y

**Published:** 2017-03-27

**Authors:** Junjing Deng, David J. Vine, Si Chen, Qiaoling Jin, Youssef S. G. Nashed, Tom Peterka, Stefan Vogt, Chris Jacobsen

**Affiliations:** 1 Applied Physics, Northwestern University, Evanston, IL 60208 USA; 2 Advanced Photon Source, Argonne National Laboratory, Argonne, IL 60439 USA; 3 Department of Physics & Astronomy, Northwestern University, Evanston, IL 60208 USA; 4 Mathematics and Computing Science Division, Argonne National Laboratory, Argonne, IL 60439 USA; 5 Chemistry of Life Processes Institute, Northwestern University, Evanston, IL 60208 USA; 6 X-ray Science Division, Advanced Photon Source, Argonne National Laboratory, Argonne, IL 60439 USA; 7 Advanced Light Source, Lawrence Berkeley National Laboratory, Berkeley, CA 94720 USA

## Abstract

X-ray microscopy can be used to image whole, unsectioned cells in their native hydrated state. It complements the higher resolution of electron microscopy for submicrometer thick specimens, and the molecule-specific imaging capabilites of fluorescence light microscopy. We describe here the first use of fast, continuous x-ray scanning of frozen hydrated cells for simultaneous sub-20 nm resolution ptychographic transmission imaging with high contrast, and sub-100 nm resolution deconvolved x-ray fluorescence imaging of diffusible and bound ions at native concentrations, without the need to add specific labels. By working with cells that have been rapidly frozen without the use of chemical fixatives, and imaging them under cryogenic conditions, we are able to obtain images with well preserved structural and chemical composition, and sufficient stability against radiation damage to allow for multiple images to be obtained with no observable change.

## Introduction

Studies of cells and tissues often require that multiple imaging methods be employed. As an example, one might want to image the distribution of a specific ion or protein type within a cell, while also gaining an overall view of cellular structure in order to provide biological context. Superresolution techniques in visible light fluorescence microscopy^1–3^ provide high resolution views of certain pre-selected molecules, while approaches such as structured illumination^4^ provide improved resolution of the unlabeled components of a cell using light microscopy. Even higher resolution views of unlabeled structure are provided using Correlative Light and Electron Microscopy (CLEM)^5–7^ as described in recent reviews^8–10^. However, in the case of electron microscopy, one is limited to images either of exposed cellular surfaces in scanning electron microscopy, or cell types or regions limited to about 1 *μ*m thickness in transmission electron microscopy^11, 12^ (thicker regions of cells can be imaged at somewhat lower resolution using scanning transmission electron microscopy^13–15^).

X-ray microscopy offers important capabilites that are complementary to the above approaches. Soft x-rays in the “water window” spectral region of 290–540 eV have been used to image whole, frozen-hydrated cells at 30 nm resolution in 2D^16^ and 30–50 nm in 3D^17–19^, while multi-keV x-rays can be used to stimulate x-ray fluorescence for studies of trace elements in cells and tissues^20, 21^ including in 3D^22^. X-ray fluorescence microscopy provides a considerable improvement in sensitivity at a given radiation dose than in electron or proton microprobes^23, 24^, so that one can quantitatively view ion distributions at physiologically meaningful concentrations. While the use of core-level excitations of ions implies high radiation dose, damage effects can be mitigated by using cryogenic specimen imaging conditions^25–27^, and cryofixation also provide less perturbation on trace element content when compared to chemical fixation^28–32^. X-ray fluorescence bypasses several problems encountered when using visible light fluorophores to study trace metals: (1) fluorophores require exact knowledge of the different binding affinities in all of the cell’s biochemical and subcellular compartments, and the knowledge that fluorophores can even enter such compartments; (2) fluorophores are available for only a subset of interesting trace metals, and (3) only certain ionic forms of trace metals are visible using any one of these fluorophores. When using multi-keV x-rays as required to stimulate x-ray fluorescence from most biologically-interesting trace elements, cells show poor absorption contrast for complementary whole-cell structural imaging; however, cells also show strong x-ray phase contrast^33^ so that several studies have combined x-ray fluorescence and phase contrast to allow for simultaneous imaging of subcellular structure and trace metal distributions^34, 35^.

While x-ray nanofocusing optics are undergoing steady resolution improvements^36, 37^, imaging based on phasing large-angle coherent x-ray diffraction data provides an alternative for transmission imaging with no lens-imposed resolution limits^38^. In particular, x-ray ptychography^39^ offers the ability to image large fields of view. In ptychography, a lens is used to focus the x-ray beam to a spot through which the specimen is scanned, with the overlap between illumination spots providing a powerful constraint for reconstruction of both the specimen transmission function and the illumination spot or probe function^40–42^. By using a pixelated area detector in the forward scattering direction to collect coherent diffraction data, and an energy-dispersive detector perpendicular to the incident beam to collect the x-ray fluorescence signal, one can obtain both ptychographic transmission images and fluorescence images of specific elemental distributions simultaneously in the same experiment^43^, and the ptychographically-recovered probe function can be used to improve the resolution of the fluorescence image through deconvolution^44^. Since x-ray ptychography delivers both absorption and phase contrast, this combined approach can be used to study both trace elements via fluorescence and overall cellular structure via phase contrast.

We have previously shown that this combined x-ray ptychography and fluorescence approach can be used to image frozen hydrated cells under cryogenic conditions^45^. We show here advances in this combined approach that lead to superior image quality, a spatial resolution of better than 20 nm in ptychography, and a 60× reduction in radiation dose (compared to our earlier experiments) to what we believe is close to the theoretical limit for this imaging method. The advances employed are a continous scanning method for faster data acquisition, a ptychographic reconstruction approach that accounts for both the new scanning approach and for partial coherence in the x-ray beam, and a signal-dependent probe deconvolution method for x-ray fluorescence images of trace element distributions at sub-100 nm spatial resolution. While several of these developments have been separately demonstrated for the imaging of microfabriated test specimens, we show here their combined application for frozen hydrated cell imaging.

## Results

**Figure 1:**
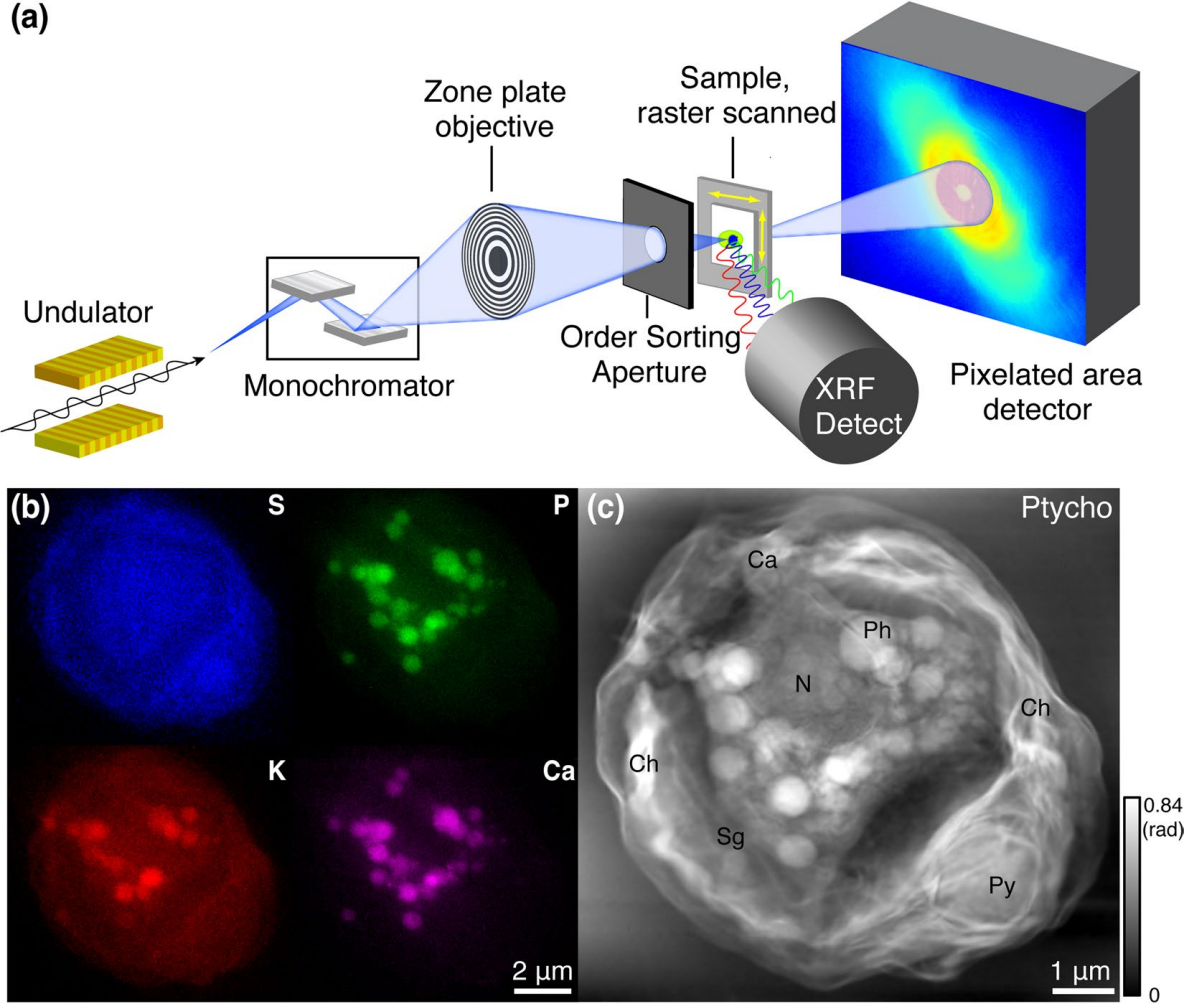
Combined x-ray fluorescence and ptychographic imaging of a frozen hydrated *Chlamydomonoas reinhardtii* alga. The x-ray microscope^27^ is shown schematically in (**a**)^45^, where an energy-dispersive detector was used to record x-ray fluorescence and a pixelated area detector was used to record coherent diffraction patterns for ptychographic image reconstruction. The x-ray fluorescence maps of the elements S, P, K, and Ca shown in (**b**) have a spatial resolution that is consistent with the ~85 nm theoretical Rayleigh resolution of the focused x-ray beam, while the phase contrast ptychographic image (**c**) shows considerably more detail due to the reconstruction of x-ray scattering at an angle well beyond the numerical aperture of the Fresnel zone plate used. This image shows a single cup-shaped chloroplast (Ch), as well as a number of other organelles: pyrenoid (Py), nucleus (N), starch granule (Sg), and polyphosphate bodies (Ph). We also note the presence of granules (labeled Ca) with high calcium and phosphorus content, but low sulfur and potassium content.

Our measurements were performed using the x-ray beamline and instrument described below in *Methods* (the same apparatus was used in our prior work on move-settle-measure ptychography of frozen hydrated specimens^27, 45^) and shown schematically in Fig. 1(a). Our prior work was limited by using a move-settle-measure sequence for collecting data from each overlapping x-ray illumination spot, with the move-settle time comprising nearly half of the experiment time so that it added to the radiation dose received by the specimen since a sufficiently fast x-ray beam shutter was not available. Following the development of multi-probe-mode ptychographic reconstruction algorithms^46^, this limitation has been overcome by using continous probe scanning in x-ray ptychography as anticipated in simulations^47^ and demonstrated experimentally on microfabricated test patterns^48–50^, though (until now) not on frozen hydrated biological specimens. The fluorescence and transmission pixel array detectors integrated signals during continuous scan motion in the horizontal (*x*) direction over position increments of 50 nm, with a corresponding pixel transit time of 100 msec. Successive horizontal scan lines were acquired at 50 nm increments in the vertical, so that we estimate that the photon density incident upon the specimen to be about 9.2 × 10^3^ photons/nm^2^ at 5.2 keV (the photon density in our previous experiments^45^ was about 5.6 × 10^5^ photons/nm^2^ due to slightly higher focused flux, smaller pixel step size, and much longer per-pixel acquisition time; see Discussion). Acquisition of a 201 × 221 pixel scan grid with a field of view of 10 × 11 *μ*m took about 75 minutes, which is about 5 times faster than step scanning over the same imaging field in the Bionanoprobe. The collected diffraction patterns were fed into an optimized ptychographic reconstruction program^51^ based on the ePIE^52^ ptychographic reconstruction algorithm (but with multi-probe-mode capabilites^46^ included) to recover the complex-valued transmission function of the sample, along with the multi-mode probe functions. Because the datasets involved are fairly large (11.2 GBytes in this case), this reconstruction code uses a graphical processing unit (GPU) for parallel processing speedup, with the capability of also using message-passing-interface (MPI) parallel programming so as to exploit multiple GPU-equipped nodes in a cluster^51^.

The green algae *Chlamydomonas reinhardtii* serves as a model cell for studying photosynthesis^53^, and has been studied in electron microscopy including via thin sections from fixed, stained, and embedded specimens^54^ and cryo electron microscopy of focused-ion-beam prepared thin regions^55^. These algae have also been studied in a frozen hydrated state using soft x-ray microscopy^16^ and tomography^56^, and hard x-ray ptychographic tomography at 180 nm resolution^57^. For our experiments, we chose 5.2 keV as the incident x-ray energy in order to excite x-ray fluorescence from the elements S, P, K, and Ca while also obtaining stronger phase contrast than would be present at higher photon energies. In Fig. 1, we show five views of such a *Chlamydomonas* alga consisting of the x-ray fluorescence images of these four elements (Fig. 1(b)) along with the ptychographically-reconstructed phase contrast image (Fig. 1(c)). Because the ptychography image includes x-rays scattered by smaller features to larger angles than the numerical aperture subtended by the Fresnel zone plate, it shows considerably higher resolution. In addition, the bulk mass of a cell is primarily comprised of the elements C, N, and O which are difficult to detect by x-ray fluorescence, but these elements show strong x-ray phase contrast in transmission imaging. We note that the confinement of potassium (K) within the cell indicates that the cell’s membrane was intact at the moment of cell plunge-freezing.

**Figure 2:**
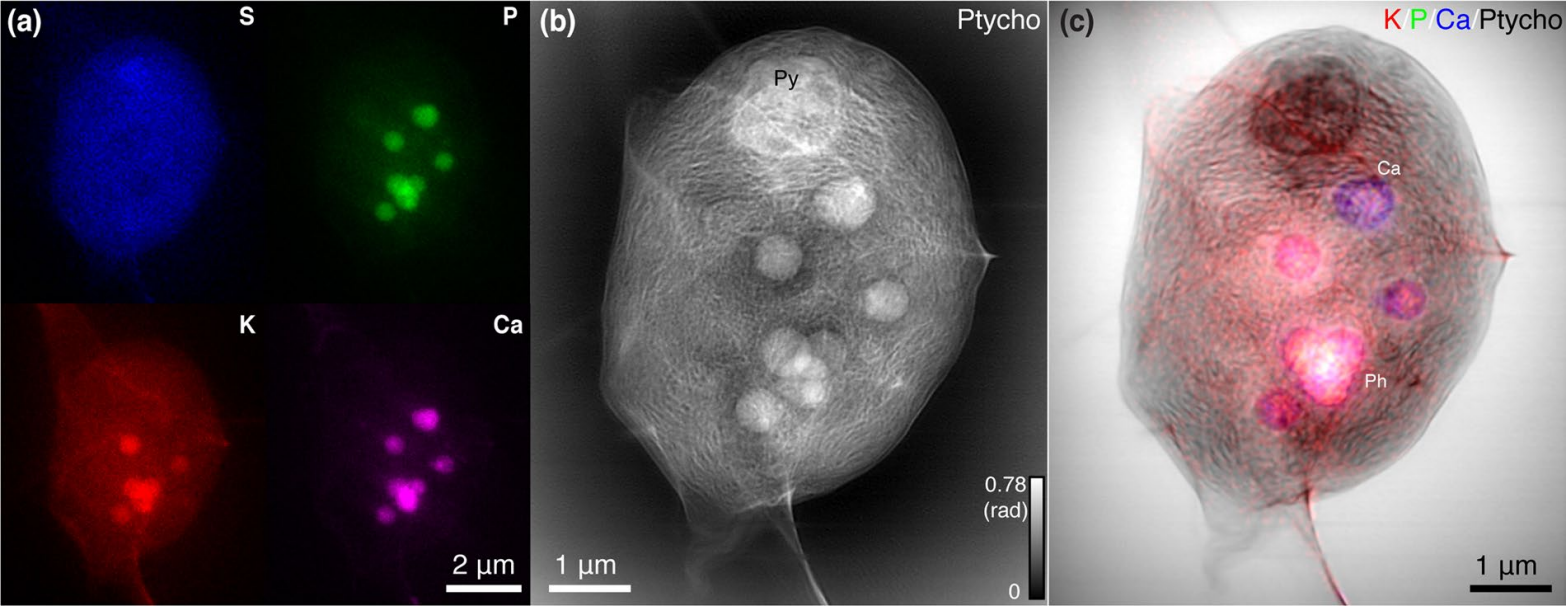
Fluorescence and ptychographic x-ray images of a second unsectioned frozen-hydrated *Chlamydomonas* alga. X-ray fluorescence images (**a**) show the distribution of S, P, K, and Ca within the plunge-frozen cell. The 5.2 keV x-ray ptychographic phase contrast image (**b**) shows unlabeled subcellular structures, including a big pyrenoid (Py) on the top. Because the fluorescence and ptychographic image data are recorded simultaneously, the various images are in perfect registry allowing for (**c**) which is a color composite image overlay of K, P, and Ca on top of the ptychographic image. With this method, the electron-dense spherical structures in (**b**) can be identified as polyphosphate bodies (Ph) that contain polyphosphate complexed with calcium^58^. Again, the granule (labeled Ca) seems to be different from other polyphosphate bodies as it contains less potassium, which suggests that this granule might undergo degeneration or aging.

Figure 2 shows the results of a combined scan on another *Chlamydomonas* alga with more fine structures revealed throughout the cell. In this case, we show the S, P, K, and Ca x-ray fluorescence images and the x-ray ptychographic image as in Fig. 1, but we also add a third image with an overlay of the K, P, and Ca images on top of the ptychograpic image. This composite image shows the benefit of the combined fluorescence and ptychography imaging approach, since all image signals were recorded simultaneously with no effort required to register the various images to each other. We are therefore able to put trace element distributions into their proper biological context (see Fig. 2(c)). The elemental information combined with ptychographic image enables the identification of what each organelle is. For example, the electron-dense spherical structures in Fig. 2(b) represent polyphosphate bodies (Ph) based on their enriched content of phosphate and calcium^58^.

**Figure 3:**
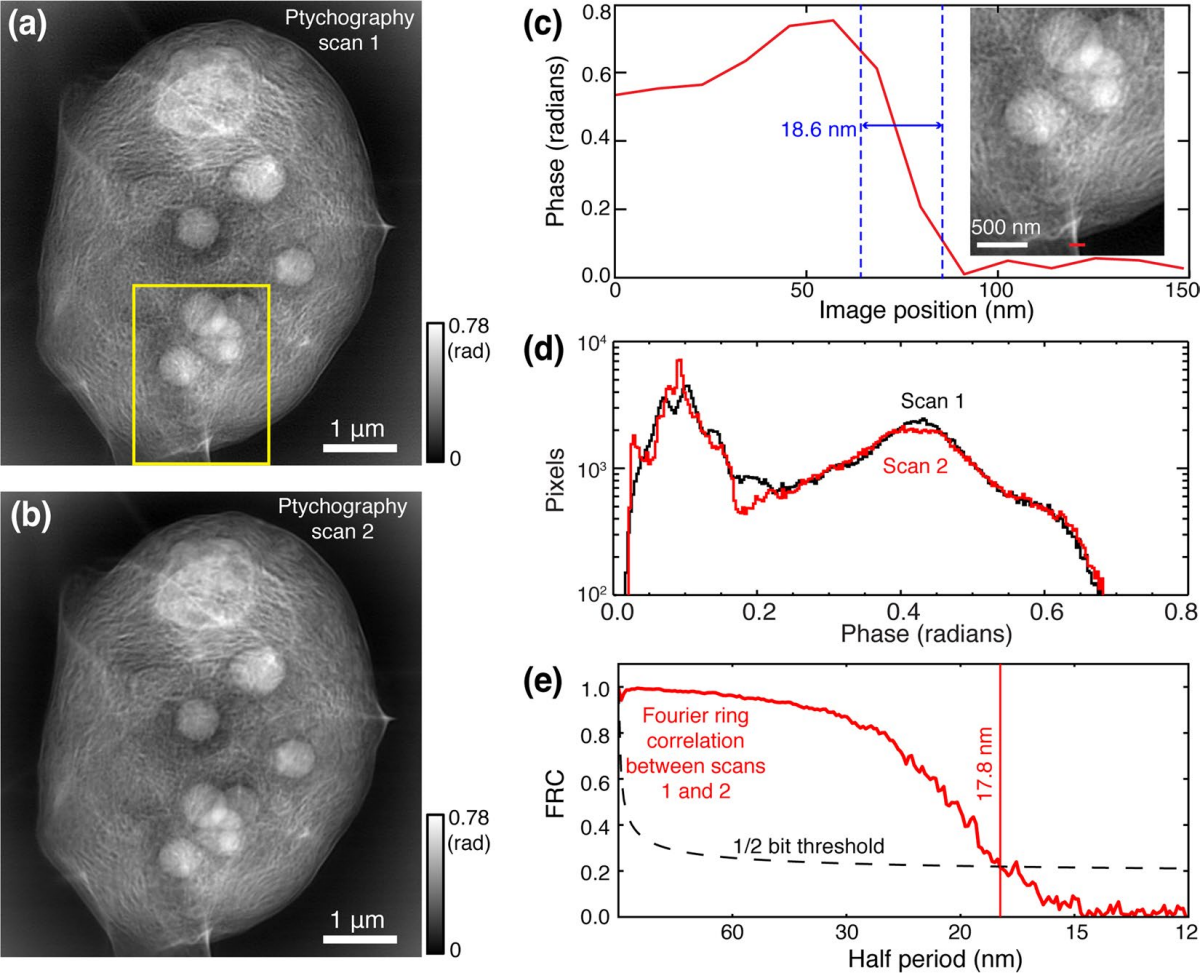
Resolution analysis of x-ray ptychographic images of a *Chlamydomonas* alga imaged in the frozen hydrated state. Two successive, independent images were obtained of the same specimen; in fact, image (**a**) is the same image as Fig. 2(b), while image (**b**) here was obtained soon after. The inset in (**c**) is a zoomed region taken from the yellow box in (**a**). The line profile indicated by a red line in the inset shows an edge response (10–90%) of 18.6 nm. Histograms of image (**a**) and (**b**) are plotted in (**d**), which shows that the images are quite consistent except at low values of optical phase (thus low electron density), which may be due to some ice evaporation. Because image (**a**) and (**b**) represent two independent images of the same object, object features should correlate while noise and other imaging flaws should not; the correlation measured as a function of spatial frequency (the Fourier ring correlation or FRC^62, 63^) is shown in (**e**) along with the accepted 1/2-bit threshold for sufficient correlation^64^. This FRC measure gives an estimated half-period resolution of 17.8 nm for this whole, hydrated eukaryotic cell.

The spatial resolution obtained by ptychography is not limited by optics, but rather by the highest-diffraction-angle signals acquired by the detector and reconstructed by the ptychographic phasing algorithm. As a result, ptychography can deliver a resolution that exceeds the lens resolution limit. In iterative phase retrieval methods, one measure of consistency of the reconstructed image is the phase retrieval transfer function^59^ (also known as a reconstructed intensity ratio^60, 61^) which measures the resolution dependence of residual fluctuations in the iterative image reconstruction process. However, a better measure of spatial resolution is provided by comparing two independently-obtained images of the same specimen obtained using the same method. Real features present in the specimen should be correlated in the two images, while fluctuations due to noise should show no correlation. Measuring the correlation of phase in the Fourier domain as a function of spatial frequency (the inverse of spatial periodicities in the image) gives rise to a Fourier ring correlation^62, 63^ for which a 1/2-bit threshold provides an accepted resolution threshold^64^. In Fig. 3(a), we show the ptychographic image shown in Fig. 2(b) as well as a second image obtained in a subsequent scan of the same specimen in Fig. 3(b). The second image shows no obvious structural changes. Comparing the histograms of these two scans (see Fig. 3(d)), they are quite consistent except at low-value range (low projected electron density), which may be due to some ice evaporation. The Fourier ring correlation between these two images shown in Fig. 3(e) crosses the standard 1/2-bit resolution threshold^64^ at a half-period of 17.8 nm, which agrees with an estimate of 18.6 nm given by line-profile analysis in Fig. 3(c).

**Figure 4:**
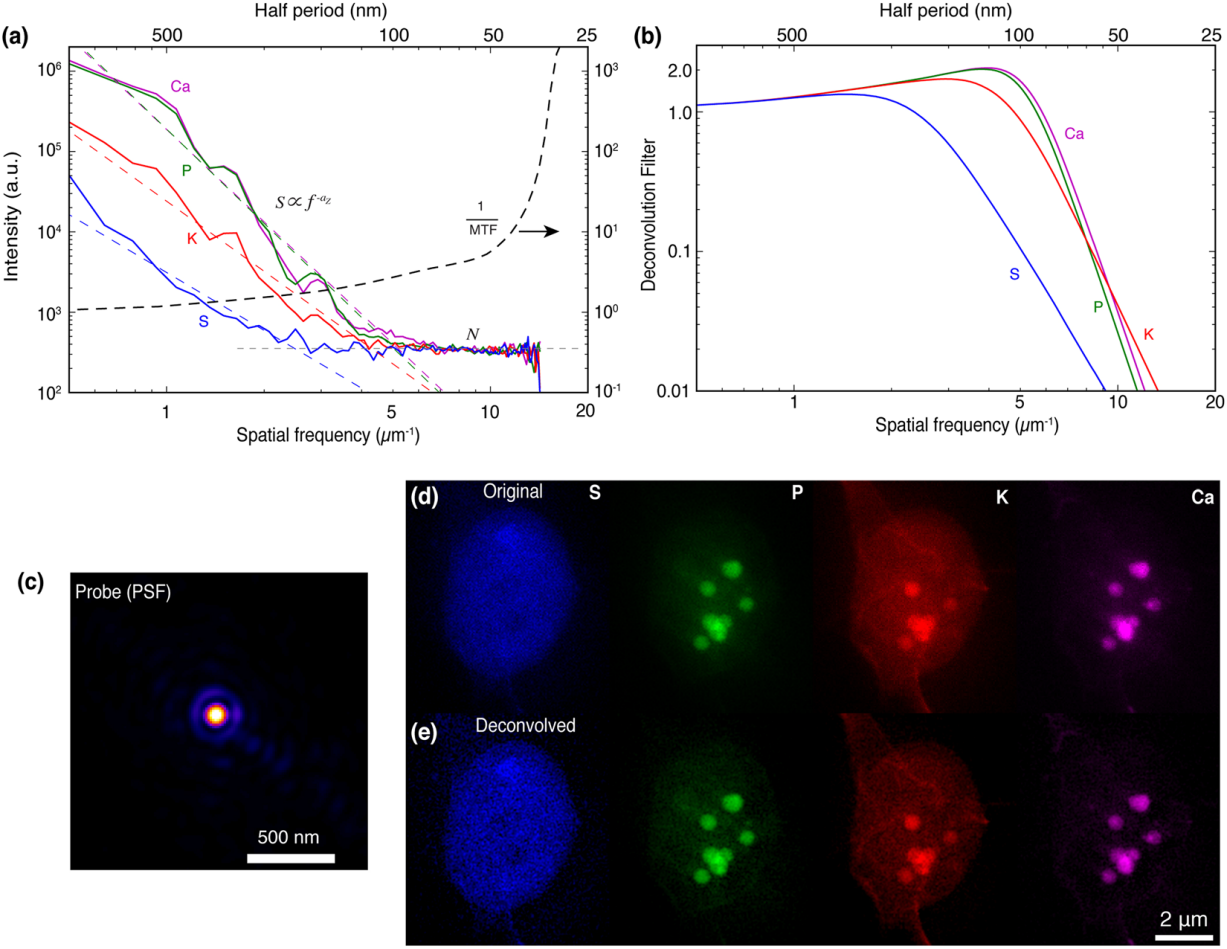
Deconvolution of fluorescence images using the reconstructed x-ray probe provided by ptychography and modified deconvolution filters. (**a**) Power spectra of Ca, P, K, S fluorescence images shown along with their respective signal trends $S\propto {f}^{-{a}_{Z}}$, and the half-period length scales *x*_cutoff_ marking the transition to a noise floor *N*. Also shown is the azimuthally-averaged inverse modulation transfer function 1/MTF(*f*) that one would use as a Fourier deconvolution filter with a noise-free image (Eq. 2). (**b**) The deconvolution filters *D*_*Z*_(*f*) for the elements *Z* = Ca, P, K, and S obtained using Eq. 4. (**c**) The intensity point spread function (PSF) of the reconstructed probe, or *p*(*x*, *y*), provided by ptychography; its inverse Fourier transform, azimuthally-averaged, leads to the modulation transfer function (MTF) plotted in (**a**) and contributes to the deconvolution filters *D*_*Z*_(*f*) in (**b**). The fluorescence maps *i*(*x*, *y*) before deconvolution are shown in (**d**) (these are the same images as shown in Fig. 2(a)), and the deconvolved images *o*(*x*, *y*) are shown in (**e**). The deconvolved images show improved detail, and a reduction of a poorly-spatially-resolved fluorescence “haze” on the images.

Unlike ptychography, the spatial resolution in x-ray fluorescence microscopy is limited by the probe size produced by the focusing optic. In fact, one can use the probe function recovered by ptychography and deconvolve it from the fluorescence images to obtain higher spatial resolution^44^. The fluorescence image *i*(*x*, *y*) is a convolution of the actual fluorescence distribution *o*(*x*, *y*) (the object) and the intensity point spread function of the probe *p*(*x*, *y*), or

1 $$i(x,y)=o(x,y)\,\ast \,p(x,y),$$

where * denotes convolution. The convolution of two functions *i*(*x*, *y*) = *o*(*x*, *y*) * *p*(*x*, *y*) can be represented in reciprocal space by the product of their Fourier transforms, or *I*(*f*_*x*_, *f*_*y*_) = *O*(*f*_*x*_, *f*_*y*_) · *P*(*f*_*x*_, *f*_*y*_) where {*f*_*x*_, *f*_*y*_} are spatial frequencies and $I({f}_{x},{f}_{y})= {\mathcal F} \{i(x,y)\}$ is used to represent a Fourier transform. As a result, the object *o*(*x*, *y*) can be recovered from the recorded image data *i*(*x*, *y*) using

2 $$o(x,y)={ {\mathcal F} }^{-1}\{\frac{I({f}_{x},{f}_{y})}{P({f}_{x},{f}_{y})}\}={ {\mathcal F} }^{-1}\{\frac{ {\mathcal F} \{i(x,y)\}}{{\rm{MTF}}({f}_{x},{f}_{y})}\},$$

where *P*(*f*_*x*_, *f*_*y*_) is the Fourier transform of the intensity point spread function which is in fact the modulation transfer function MTF(*f*_*x*_, *f*_*y*_). Starting from the complex probe modes recovered along with the ptychography image of Fig. 2(b), we calculated their image-wide average weighting to generate a net intensity point spread function *p*(*x*, *y*) shown in Fig. 4(c) and its azimuthally-averaged inverse modulation transfer function 1/MTF(*f*) shown in Fig. 4(a).

The deconvolution method of Eq. 2 fails when noise is present at high spatial frequencies, which is almost always the case. In our example, the low concentration of trace elements, and the secondary processes that lead to a detected x-ray fluorescence signal (absorption “jump ratio,” fluorescence yield, and detector solid angle^24, 65^), cause the high spatial frequency signal levels in x-ray fluorescence images to be obscured by quantum noise. If one examines the square of the Fourier transform of an image as a function of spatial frequency *f*, one obtains a power spectrum over which the signal declines as *f* ^−*a*^ (where *a* ≃ 3–4 in many cases) until one reaches a half-period length scale *x*_cutoff_ where the power is dominated by spatially uncorrelated fluctuations. Because these quantum noise fluctuations are uncorrelated pixel-to-pixel, they have the spatial characteristic of discrete delta functions which in turn have a “flat” or constant power spectrum^66^. In Fig. 4(a) we show power spectra calculated from the x-ray fluorescence images shown in Fig. 2(a). For each element *Z*, we carried out a linear fit on a log-log scale to determine the power law dependence factor *a*_*Z*_, and the half-period length scale of *x*_cutoff,*Z*_ marking the transition to a “flat” noise floor. The respective values of these two parameters are *a*_S_ = 2.39 and *x*_cutoff,S_ ≃ 130 nm, *a*_K_ = 2.90 and *x*_cutoff,K_ ≃ 110 nm, *a*_P_ = 3.89 and *x*_cutoff,P_ ≃ 100 nm, and *a*_Ca_ = 3.81 and *x*_cutoff,Ca_ ≃ 100 nm. A similar power spectrum analysis on fluorescence images was reported earlier^67^.

One way to incorporate this simple signal-versus-noise characterization is to incorporate a Weiner filter^68^ into the deconvolution process^67, 69, 70^. The Wiener filter *W*(*f*_*x*_, *f*_*y*_) of

3 $$W({f}_{x},{f}_{y})=\frac{|S({f}_{x},{f}_{y}){|}^{2}}{|S({f}_{x},{f}_{y}){|}^{2}+|N({f}_{x},{f}_{y}){|}^{2}}.$$

is an optimal filter if one has *a priori* knowledge of the spatial frequency distribution of the signal *S*(*f*_*x*_, *f*_*y*_) and noise *N*(*f*_*x*_, *f*_*y*_) powers; in our case, we have good estimates of $S\propto {f}^{-{a}_{Z}}$ and *N* = constant as shown in Fig. 4(a). The Wiener filter can be combined with the modulation transfer function to lead to a combined deconvolution filter *D*(*f*_*x*_, *f*_*y*_) of

4 $$D({f}_{x},{f}_{y})=\frac{W({f}_{x},{f}_{y})}{{\rm{MTF}}({f}_{x},{f}_{y})}$$

in which case Eq. 2 becomes

5 $$o(x,y)={ {\mathcal F} }^{-1}\{ {\mathcal F} \{i(x,y)\}\cdot D({f}_{x},{f}_{y})\}$$

for recovery of the fluorescing object *o*(*x*, *y*) from the measured fluorescence intensity *i*(*x*, *y*). These deconvolution filters are shown in Fig. 4(b), and they indicate that the stronger fluorescence signal from elements Ca, P, and K should allow for a higher resolution recovered object *o*(*x*, *y*) than will be possible from the weaker sulfur (S) fluorescence signal. This is indeed seen in Fig. 4(e), where the deconvolved fluorescence images *o*(*x*, *y*) are shown alongside the as-recorded fluorescence images *i*(*x*, *y*) in Fig. 4(d). To our knowledge, this is the first demonstration of a systematic approach to incorporating element-specific signal characteristics into deconvolution of a ptychographically-recovered probe function from x-ray fluorescence images.

## Discussion

We have shown for the first time the application of fast, continuous scanning x-ray ptychography and fluorescence microscopy to high resolution imaging of frozen hydrated cells. The use of these two imaging modalities in one single experiment allows us to obtain complementary information on cells that were rapidly frozen from the living state: structural information at sub-20-nm resolution by ptychography, and x-ray fluorescence measurements of element distributions at ~100-nm resolution. We have also shown that the ptychographically-recovered probe function can be deconvolved from the fluorescence images to improve their resoution; while has been demonstrated previously^44^, we present here a systematic approach to use the actual signal and noise levels in the fluorescence images to optimize the deconvolved images of each element’s distribution.

As noted in Results above, this present work exposed the specimen to a detected fluence about 60× lower than in our previous work^45^, or 9.2 × 10^3^ photons/nm^2^ as opposed to 5.6 × 10^5^ photons/nm^2^. At a photon energy of 5.2 keV, and assuming a stochiometric composition of protein to be^71^ H_48.6_C_32.9_N_8.9_O_8.9_S_0.6_ with a condensed density of 1.35 g/cm^3^, the 1/*e* linear attenuation length for x-ray absorption in protein can be calculated from tabulated x-ray optical constants^72^ to be 247 *μ*m. This means that the estimated radiation dose imparted to condensed protein was reduced from about 1.8 × 10^9^ Gray to about 2.9 × 10^7^ Gray (in both cases this estimated dose to the specimen includes the 24% loss in a Be window and an air gap, whereas the image statistics are based on the *detected* signal rather than the signal incident upon the specimen), yet higher image quality and improved resolution was obtained. At the higher dose used previously, it may have been that fine cellular structure was being degraded during the course of the exposure, since it is known in protein crystallography that high resolution diffraction spots begin to fade at a critical dose of about 2 × 10^7^ Gray^73^. (Because x-ray microscopy requires only the preservation of density at 10–20 nm length scales, the fading of diffraction spots arising from atomic bonds may not be the relevant criterion, and there is some evidence that higher doses can be tolerated in x-ray microscopy^16, 74^). It is also possible that our earlier experiment suffered from losses in image quality and resolution due to partial coherence in the x-ray probe beam, which can be separately factored out from the image in multi-probe-mode ptychography reconstructions^46^ such as we used^49^.

Is this reduced fluence value reasonable for sub-20 nm resolution x-ray ptychographic imaging of cells? To estimate the photon exposure required, we calculated the normalized image intensity *I*_*f*_ in areas containing feature material (20 nm thick protein, with a x-ray refractive index estimated^72^ to be *n* = 1 − *δ* − *iβ* = 1 − 1.11 × 10^−5^ − *i*7.70 × 10^−8^) versus the intensity *I*_*b*_ in areas containing background material (20 nm amorphous ice at a density of 0.92 g/cm^3^, with *n* = 1 − *δ* − *iβ* = 1 − 7.92 × 10^−6^ − *i*6.60 × 10^−8^). In order to estimate the image intensities *I*_*f*_ and *I*_*b*_, we used an expression for Zernike phase contrast^75^ in the thin feature limit, ignoring absorption in the Zernike phase ring; since ptychography delivers a phase contrast image, we assume that it follows similar characteristics. Both image intensities were then assumed to be reduced by an overlying thickness *t*_overlayer_ = 2 *μ*m characterized by a 1/*e* attentuation length of ${\mu }_{{\rm{overlayer}}}^{-1}=\lambda /\mathrm{(4}\pi \beta )$ which is 287 *μ*m for amorphous ice at 5.2 keV. Once these intensities are calculated, if one assumes that limited photon statistics sets the noise limit and uses a Gaussian approximation to the Poisson distribution, the number of photons $\bar{n}$ per pixel required for imaging a feature against a background with a given signal-to-noise ratio (SNR)^76, 77^ is found from

6 $$\bar{n}=\frac{{{\rm{SNR}}}^{2}|{I}_{f}-{I}_{b}{|}^{2}}{{I}_{f}+{I}_{b}}.$$

It is conventional to use the Rose criterion of SNR = 5 based on studies of human image perception^78^ (although lower values of SNR are often accepted in electron microscopy). This gives rise to an expression for the required exposure $\bar{n}$ of photons required to see a *t* = 20 nm thick feature in a pixel of

7 $$\bar{n}\simeq \frac{25}{8{\pi }^{2}}\frac{{\lambda }^{2}}{{t}^{2}}\frac{1}{|{\delta }_{f}-{\delta }_{b}{|}^{2}}\exp [2{\mu }_{{\rm{overlayer}}}{t}_{{\rm{overlayer}}}]$$

from which we obtain an estimate of $\bar{n}=4.51\times {10}^{6}$ photons per (20 nm)^2^ pixel area, or 11.3 × 10^3^ photons/nm^2^. Of course this estimate makes many assumptions including of high efficiency for recording the x-ray diffraction pattern (reasonable since helium gas was used to fill the flight tube from the Bionanoprobe to an efficient pixel array detector), so one should not place too much emphasis on the very strong numerical agreement of this result with our experimentally detected fluence of 9.2 × 10^3^ photons/nm^2^. However, it does indicate that the resolution of our images is reasonable given the applied exposure.

As noted above, the continuous-scanning mode method of data acquisition allowed us to increase the speed of data collection by a factor of 5. This speedup factor can be improved further using smaller exposure times with higher brightness sources and faster detectors^79^. The fast data collection is followed by rapid data processing using a GPU-based parallelized reconstruction code^51^, which allows experimenters to do on-site real time data analysis during their experiments, thus enabling increased throughput in ptychographic imaging without lens-imposed resolution limits.

## Methods

### Frozen-hydrated sample preparation

*Chlamydomonas reinhardtii* (about 10 *μ*m) were grown mixotrophically in a Tris-Acetate-Phosphate medium at 296 K on a rotary shaker (100 rpm). Five microliters of fresh cell suspensions were dispersed on Si_3_N_4_ windows (200 nm thick, 1.5 × 1.5 mm membrane area) and incubated for 10 minutes in a humidified petri dish. In order to produce a reasonably thin (a few microns) water layer with minimal ice crystal formation, cell suspensions were blotted prior to plunge freezing. In the blotting process, the windows were mounted on the Vitrobot, where the chamber temperature was set at 20 °C, and the humidity was set to be 100%. When the sample window was brought into the chamber, a pair of filter papers was used to remove extra liquid with a blot time of 2 seconds and a blot offset of 0 mm. After blotting, the sample window was plunged into the liquid ethane bath cooled by liquid nitrogen. The frozen-hydrated cells on Si_3_N_4_ windows were transferred from liquid ethane to liquid nitrogen, where we used a cryogenic light microscope with a 0.45 NA objective to observe the samples prior to x-ray microscopy.

### X-ray beamline and microscope

Our measurements were carried out at the Bionanoprobe^27^, a hard x-ray fluorescence nanoprobe with cryogenic sample environment that located at beamline 21-ID-D of the Advanced Photon Source at Argonne National Laboratory. A monochromatic x-ray beam at 5.2 keV photon energy was focused by a Fresnel zone plate with an outermost zone width of 70 nm (with a theoretical Rayleigh resolution of 85 nm); the focused x-ray flux was 2.3 × 10^8^ photons/sec as measured with a calibrated photodiode just outside the chamber (due to 15% absorption in a Be window, and about 5% absorption in air, the flux on the specimen was about 24% higher). The Bionanoprobe uses a robotic specimen exchange system to load cartridge-mounted specimens onto a piezo-driven scanning stage, with the specimen maintained at a temperature below 110 K in the ~10^−7^ torr vacuum environment of the microscope. Fluorescence signals and ptychographic diffraction patterns were simultaneously recorded in one experiment: as the sample was raster-scanned, a collimated four-element silicon drift detector (Vortex-ME4) mounted at 90 degrees to the incident x-ray beam was used to collect fluorescence spectra, while far-field diffraction patterns were acquired with a Pilatus 100 K detector at a distance of 2.1 meters downstream the sample (with a He-filled tube in between to reduce air scattering and absorption). The scan field positions are measured using a laser interferometer so that the majority of pixels have position consistency of better than 15 nm (see Fig. 7 of ref. 27).

The above apparatus is the same as had been used for our previous frozen hydrated specimen studies^45^ where we used a move-settle-measure or step-scan mode of data collection. We also used the Bionanoprobe for developing the continuous or “fly-scan” data collection technique^49^, but in that case we used nanofabricated test specimens imaged at room temperature. The fast scan was set along the horizontal (*x*) axis, where the piezo scanning stage was set to move across the a scan line at a constant velocity by a Delta Tau Turbo PMAC2 Ultralite VME motion control system; this control system used feedback provided by a laser interferometer system reading both zone plate and piezo scanning stage positions to match the desired position versus time to an accuracy of several nanometers^27^.

### Data acquisition and reconstruction

As the sample was scanned through the probe with continuous motion in the horizontal (*x*) direction, the fluorescence signals and diffraction patterns were collected simultaneously over position increments of 50 nm, with a corresponding pixel time of 100 msec (96 msec exposure time and 4 msec data readout time). Reconstructions were performed by a computer code employing graphical-processing-units (GPUs) to speed up data processing^51^. This code also uses the multiple mode reconstruction method^46^ to reconstruct both the object and several probe modes from diffraction patterns acquired in fly-scan mode. The central 256 × 256 of each diffraction pattern were cropped for ptychography reconstruction, resulting in a pixel size of about 11.4 nm in the real space.
